# The Placenta, the Organic Nervous System, the Blood, the Oxygen, and the Animal Nervous System, Physiologically Examined

**Published:** 1862-01

**Authors:** 


### Book Notices.

THE PLACENTA, THE ORGANIC NERVOUS SYSTEM, THE BLOOD, THE OXYGEN, AND THE ANIMAL NERVOUS SYSTEM, PHYSIOLOGICALLY EXAMINED. — By JOHN O'REILLY, M. D., Licentiate and Fellow of the Royal College of Surgeons in Ireland, Resident Fellow of the New York Academy of Medicine, etc., etc. New York: S. S. & W. WOOD, 389 Broadway. 1861.

The several papers, of which this volume is composed, have been, heretofore, noticed in our pages, and have attracted a considerable share of the attention of the profession; not less from their unique literary composition and arrangement, than from the intrinsic merit and originality of the views therein presented. The subjects treated of, possess an absorbing interest and importance to the physiologist; and, although the propositions Dr. O'REILLY offers, are startling in their novelty and boldness, he has presented such an array of facts and authorities, has evidently expended so vast an amount of research and thought, that they claim more than consideration,—investigation; and the student in this domain, cannot but thank him for the many interesting paths he has pioneered for further exploration. Every page is replete with some new view, some unlooked-for grouping of recondite and apposite facts; and these analogies, so novel and yet so correct, in most cases, furnish him with the most interesting and,—if true,—the most momentous deductions and inferences.

*E. g.*; the placenta, he compares to the liver in its anatomical organization and function,—the hepatic arteries in this, being analagous to the maternal uterine in that; the vena porta to the hypogastric artery of the fœtus; the hepatic veins to the branches of the umbilical veins, and the gall-ducts to the uterine veins. This analogy is made more complete by tracing a correspondence in the contents of these vessels, in their nerve-supply, and in their anatomical organization and office. By a series of such premises and comparisons, he arrives at the proposition: That the blood is arterialized by the combined influence of the organic nerves derived from the mother and fœtus, in the placental lobule or gland;—in the same manner that the nerves supplying other glandular bodies affect the blood in such glands, as shown by M. Bernard.

We have not space for a review, *in extenso*, of the remaining sections, and less than this would do the author gross injustice; we, therefore, refer our readers to the volume itself, for Dr. O'Reilly's views of the organic nervous system, etc., etc., the first of which is an ultra-recognition of the doctrine, that *Life is Nerve-action*, and its seat is in the organic nervous system.

